# Gene polymorphism of 3'APO-VNTR in Egyptians with coronary artery disease

**DOI:** 10.5937/jomb0-30616

**Published:** 2021-09-03

**Authors:** Rehab Elmougy

**Affiliations:** 1 Mansoura University, Faculty of Science, Chemistry Department, Division of Biochemistry, Mansoura, Egypt

**Keywords:** coronary artery disease, Apo B, dyslipidemia, genetics, PCR, koronarno arterijsko oboljenje, Apo B, dislipidemija, genetika, PCR

## Abstract

**Background:**

Coronary artery diseases (CAD) are big health problem in both developed and developing countries. It is considered one of the main causes of death in the world. Dyslipidemia increases the risk of CAD incidences. It is aimed in this worktop study the impact of 3'APOBVNTRgene on CAD incidences.

**Methods:**

Eighty CAD patients and ninety-three healthy volunteers are enrolled in this study. Lipid parameters were estimated in both groups and PCR technique has been performed to analyze 3'APOB-VNTR gene polymorphism.

**Results:**

The genotypes 31/31, 31/37, 37/37 and 31/44 are more predominant in both groups. The frequency of 24/31 in CAD patients is (0.137) while it is completely absent in the control group. Our results show that there is an increase in the frequency of various genotypes (e.g., 17/31 and 21/34 genotypes) in the control group compared to theca patients group.

**Conclusions:**

3'APOB-VNTR gene could probably be considered a risk factor for CAD incidences and may help to early diagnose them.

## Abstract (sr)

**Uvod:**

Koronarno arterijsko oboljenje (CAD) je veliki zdravstveni problem kako u razvijenim tako i u nerazvijenim zemljama. Smatra se da je jedan od glavnih uzroka smrtnosti u svetu. Dislipidemija povećava rizik od učestalosti CAD. Cilj ovog rada je bio ispitivanje uticaja 3'APOB-VNTR gena na učestalost CAD.

**Metode:**

Ovo poučavanje obuhvatilo je osamdeset CAD pacijenata i devedeset tri zdrava volontera. U obe grupe određivani su lipidni parametri a primenom PCR tehnike analiziran je genetski polimorfizam 3'-APOB-VNTR.

**Rezultati:**

U obe grupe bili su predominantni geni 31/31, 31/37, 37/37 i 31/44. Učestalost 24/31 u CAD pacijenata je bila 0,137, dok je u potpunosti izostala kod kontrolne grupe. Naši rezultati pokazuju da postoji povećanje u učestalosti različitih genotipova (npr. 17/31 i 21/34) u kontrolnoj grupi u poređenju sa grupom pacijenata.

**Zaključak:**

3'APOB-VNTR GEN može se najverovatnije smatrati kao faktor rizika za učestalost CAD i može se koristiti za ranu dijagnozu.

## Introduction

Cardiovascular diseases are a collection of heart- and blood vessels-related diseases that are globally common. Among them are coronary artery diseases that are considered one of the main causes of mortality on the globe [1] [2]. Hypertension, diabetes mellitus and obesity are the most traditional risk factors for CAD. Moreover, the accumulation of lipids (e.g., cholesterol and triglycerides due to some environmental or genetic factors) in blood is a principal cause of CAD incidences [3]. Also, there is a strong relationship between cardiomyopathy and dyslipidemia [4]. The literature shows that controlling cholesterol and triglyceride in blood might be helpful in reducing the individuals' susceptibility to the disease [5].

Apolipoprotein B (ApoB) is an important component of lipoproteins (LDL- and VLDL-cholesterol) [6] LDL is responsible for carrying cholesterol to cells that need it [7]. A strong correlation between the levels of ApoB and LDL indicates that ApoB may be a main cause of CAD incidences. The ApoB gene is located on the chromosome 2 [8] [9]. Different polymorphisms are reported at the ApoB gene. One of these polymorphisms is a variable number of tandem repeats (VNTR) at the 3' end. The polyadenylation and processing of the mRNA transcript of ApoB-100 are thought to be influenced by 3'APOB-VNTR [10].

The present work aims to study the impact of 3'APOB-VNTRon the incidence of CAD in Egyptian population.

## Materials and Methods

Eighty CAD patients with mean (±standard deviation) age 53.5 (±9.0) years and ninety-three healthy participants, whose mean (±standard deviation) age is 45.1 (±7.1) years, were included in the present study. Subjects having either hepatitis B or hepatitis C virus were excluded from the study. None of the control subjects had any signs or symptoms suggesting CAD. Based on medical history and CAD questionnaire, all control subjects were free from any other diseases.

*Blood samples.* The local Ethics Committee approved the study and informed consent was obtained from each participant. Fasting whole blood samples (5 mL) were collected from the subjects under investigation (patients and controls). The blood sample was divided into two parts, one part delivered to plain tubes and allowed to clot for 10 min, centrifuged and the separated serum was used to analyze the levels of cholesterol, triglycerides and HDL-cholesterol. The second part of the whole blood was taken in EDTA coated tubes for DNA extraction. PCR technique on these DNA samples was applied.

*Biochemical analyses*. Total cholesterol [11] and triglyceride [12] [13] were determined by standard enzymatic methods. High-density lipoprotein (HDL) was assayed by selective precipitation with phosphotungestic acid-magnesium chloride [14]. VLDL-cholesterol level has been calculated using the formula of Tunali and Yanardag [15], while LDL-cholesterol level and atherogenic index have been calculated using the formula Muruganandan et al. [16] as follows

(1) }{}VLDL\ cholesterol=\frac{Triglyceride}{5}

(2) }{}LDL\ cholesterol=Total\ cholesterol - HDL\ cholesterol - \frac{Triglyceride}{5}

(3) }{}Atherogenic\ index= \frac{Total\ cholesterol – HDL\ cholesterol}{HDL\ cholesterol}

*Determination of genotypes*. Genomic DNA was extracted from whole blood using Qiagenspin columns.

*Determination of 3'APOB-VNTR*. The amplification of 3'APOB-VNTRby PCR was carried out using a forward and reverse primer. The forward primer sequence was 5'-ATGGAAACGGAGAAATTATG-3', while the reverse primer sequence was the 5'-CCTTCTCACTTGGCAAATAC-3'. Each reaction was done with 100 ng of genomic DNA, 1.0 μL of each primer (20 pmol), 5 μL of 10x buffer solution, 4 μL dNTP and 1.0 μL (2 U) Taq polymerase in a total volume of 50 μL. The PCR program for the amplification has been executed according to that described in Ruixing et al. [17].

*Genotyping of the 3'APOB-VNTR polymorphism*. 15 μL of amplified DNA were run on agarose gel to separate the 3' VNTR alleles. The gel was stained with ethidium bromide and visualized using UV. The formula used in the calculation of the number of tandem repeats is: repeat number= (fragment length (bp)-138 bp)/15 bp [17].

*Statistical analysis*. Statistical analysis was performed with the statistical discovery software JMP 9. Quantitative data were presented as mean and SD. Qualitative data were presented as frequencies. Oneway analysis of variance (ANOVA) test was used to compare variables with a normal distribution and significance was accepted at P<0.05.

## Results

Table 1 shows that there is a highly significant increase in the levels of cholesterol, triglycerides, LDL-cholesterol, VLDL-cholesterol and atherogenic index in CAD patients in comparison with their levels in the control group. However, a significant decrease has been found in the level of HDL-cholesterol in patients compared to control subjects.

**Table 1:** Demographic characteristics of both CAD cases and healthy volunteers *SD = Standard deviation, TG = Triglyceride, HDL = High density lipoprotein, LDL = Low density lipoprotein, VLDL = Very low density lipoprotein.

Parameter	Control Mean ± SD	CAD cases Mean ± SD	p-value
Age (years)	45.1 ± 7.1	53.5 ± 9.0	
Total cholesterol (mmol/L)	7.11 ± 1.28	11.94 ± 3.50	< 0.0001*
TG (mmol/L)	2.39 ± 0.36	9.66 ± 5.81	< 0.0001*
HDL-cholesterol (mmol/L)	2.15 ± 0.29	2.75 ± 1.62	0.101
VLDL-cholesterol (mmol/L)	0.48 ± 0.07	1.93 ± 1.16	< 0.0001*
LDL-cholesterol (mmol/L)	3.78 ± 1.82	7.34 ± 3.52	< 0.0001*
Atherogenic index	2.35 ± 0.6	5.1 ± 4.3	0.0049

The frequency of ApoB genotypes in both groups (patients and controls) is shown in Table 2. The genotypes 31/31, 31/37, 37/37 and 31/44 are more predominant in both groups.

**Table 2:** Distribution of ApoB genotypes

Genotype	Control subjects		CAD Patients	
	Number observed	Frequency	Number observed	Frequency
17/17	1	0.0108	0	0
17/24	0	0	2	0.025
17/31	13	0.1398	0	0
21/21	0	0	1	0.0125
21/27	0	0	1	0.0125
21/31	2	0.0215	0	0
21/34	8	0.0860	0	0
21/37	2	0.0215	0	0
24/24	2	0.0215	3	0.0375
24/31	0	0	11	0.1375
24/44	0	0	1	0.0125
27/27	0	0	1	0.0125
27/31	1	0.0108	0	0
27/34	0	0	3	0.0375
31/31	8	0.0860	22	0.275
31/34	1	0.0108	0	0
31/37	19	0.2043	14	0.175
31/44	18	0.1935	11	0.1375
31/51	1	0.0108	1	0.0125
34/34	4	0.0430	0	0
37/37	10	0.1075	9	0.1125
37/44	2	0.0215	0	0
44/44	1	0.0108	0	0

Table 3 demonstrated that there is a highly significant decrease in the frequency of the alleles 17, 21 and 34 in CAD patients in comparison with the control subjects, while there is a highly significant increase in the frequency of the alleles 24, 27 and 31 in CAD patients when compared with the control volunteers.

**Table 3:** Frequency distribution (percent) of ApoB alleles in normal control and CAD subjects OR = Odds ratio, CI = Confidence interval

Allele	Control subjects N (%)	CAD cases N (%)	Chi-square value (p-value)	OR (95% CI)
17	15 (8.06)	2 (1.25)	21.244(< 0.0001*)	0.0815(0.019 – 0.35)
21	12 (6.45)	3 (1.88)	14.5(0.0001*)	0.142(0.042 – 0.49)
24	4 (2.15)	20 (12.50)	15.18(< 0.0001*)	6.5(2.17 – 19.45)
27	1 (0.54)	6 (3.75)	4.85( 0.0277*)	7.21(0.86 – 60.5)
31	71 (38.17)	81 (50.63)	5.42( 0.0199*)	1.66(1.08 – 2.55)
34	17 (9.14)	3 (1.88)	9.3( 0.0023*)	0.19(0.055 – 0.66)
37	43 (23.12)	32 (20.00)	0.4940.482	0.83(0.5 – 1.4)
44	22 (11.83)	12 (7.50)	1.850.174	0.6(0.29 – 1.26)
51	1 (0.54)	1 (0.63)	—	—

## Discussion

CAD is a common health problem all over the world [18]. The World Health Organization reported that CAD are the main causes of death for about 17.3 millions [19]. There are many risk factors for CAD incidence such as hyperlipidemia, diabetes mellitus and hypertension [20] [21]. Here, we are interested in studying ApoB gene polymorphism as a risk factor for CAD incidences due to its strong relation to LDL-cholesterol.

Our results show the levels of cholesterol, triglycerides, LDL-cholesterol and VLDL-cholesterol and the atherogenic index are significantly high in CAD patients compared to the control group. This finding is in line with many previous studies [22] [23]. The results show further that there is a significant increase in the frequency of various genotypes (like 17/31 and 21/34 genotypes) in the control group (with frequencies 0.1398 and 0.0860) than in CAD patients group (with zero frequencies). On the contrary, the frequency of 24/31 genotype in CAD patients group is 0.137, while it completely vanishes in the control group. These results may help in the early prediction of CAD. To our best, this is the first study carried out in Egypt that focuses on investigating 3'APOB-VNTR polymorphism in Egyptian patients with CAD.

Our finding has some similarities with the results reported by Sajib et al. [24] who conducted their study on Bangladeshi patients with type 2 diabetes mellitus. The difference might be referred to the population ethnicity or the disease. In China, Hu et al. [25] reported that the genotype and allele distributions of ApoB variants in Chinese children with primary nephrotic syndrome were not significantly different from the control volunteers.

There is a highly significant increase in the frequencies of some alleles (e.g., 24, 27 and 31 alleles) in ApoB gene in CAD patients group when compared to their correspondences in the control group. These results may support the idea that the accumulation of bad cholesterol particles in blood may attitude to genetic factors such as ApoB gene and the priority to control the blood lipids levels by different ways such as control the diet, doing physical exercises and finally administration of some drugs, which may help reduce fat particles in blood.

Acknowledgements. The author would like to thank the anonymous referees as well as the editor for their invaluable comments that helped to improve the paper.

## Conclusion

3’APOB-VNTRgene might be considered a risk factor in CAD incidence and might help to early diagnose CAD.

## Conflict of interest statement

All the authors declare that they have no conflict of interest in this work.
